# Reminiscences of the Cholera in 1832; Its Pathology and Treatment

**Published:** 1849-02

**Authors:** W. W. Reid

**Affiliations:** Rochester, N.Y.


ART. III.—*Reminiscences of the Cholera in* 1832; *its Pathology and Treatment.* By W. W. Reid, M. D.

It is not our intention to write a treatise on cholera, but simply to give a brief sketch of its incursion into this State, and of its characteristic, or more prominent phenomena. From these we hope to be able to determine its true pathology, and from thence deduce a rational mode of treatment. Much has been written on cholera, and very divers and contradictory methods of treatment recommended. How could it be otherwise? How could we have fixed principles of treatment, when most writers admit that we know nothing of "the nature," or pathology of this disease? During the prevalence of the Epidemic, in the haste, anxiety, excitement and confusion that occupied all minds, most practitioners were, in some sense, compelled to prescribe for *symptoms;* each one saw from a different point of view, and therefore adopted different remedies, and about the same success attended all. Hence, *sequences* were mistaken for *consequences.* It was only after the Epidemic had ceased, and all excitement subsided, that we had time to look back, carefully collect and compare the facts, and thus distinguish the *real* from the *apparent.*

The winter of 1831–2 was one of the coldest known for many years in Western New York. The spring was also cold and late. The diseases prevalent during the winter and spring in Rochester, were measles, scarlet fever, and small pox. As the season advanced, our usual Intermittents made their appearance, presenting at first no uncommon features. But about the first of June, they began to be attended by a troublesome looseness of the bowels. And many cases occurred that were ushered in by violent vomiting and purging, amounting to cholera morbus. When this was arrested, reaction, or fever, followed, and the succeeding paroxysms would be attended by the ordinary chill. By degrees our Intermittents disappeared, and diarrhœa and cholera morbus usurped their place. This was during the latter part of June and the first of July. Early on the morning of the 12th of July, a stranger direct from Albany, via the canal, arrived in town. He had been sick *three days with diarrhœa.* He took some pills composed of mandrake root and extract of butternut bark, prescribed by a Botanic Doctor. He was soon after seized with vomiting and purging, and died before 10 o'clock at night. On the 14th, two other strangers from abroad, were taken sick, and died the same day. No more cases occurred till the 23d, when two of our own citizens were taken and died. From this time, till the 16th of August, the Epidemic increased in violence and fatality, when it began to decline, and on the 7th of Sept. the Board of Health reported that it had subsided. But during the whole season diarrhœa and *cholera morbus* were rife, running "pari passu," increasing and declining with the Epidemic Cholera, and did not entirely disappear till October. The same condition of things existed in other cities and villages, and to some degree, in the country generally.

The Quebec Board of Health made their first Report on the 11th of June, 1832, from which it appears, that the Epidemic broke out at Grosse Isle and Quebec on the 9th inst. The Montreal Board reported on the 13th, that there had been fifteen cases, and seven deaths, in that city. At this time it was prevalent in St. Johns, Sorrel, Laparre, and Prescott. Thus, in four days time having invaded all those places, as was supposed. But some of the physicians of Montreal, assert that there had been several cases as early as April.

The New York Board first reported on the 4th of July, and from this we learn that the first fatal case was on the 27th of June. In subsequent reports we are informed that diarrhœa, and, what was considered at the time, *Cholera Morbus*, prevailed extensively throughout the city, The United States Gazette of the 4th of July, mentions "the report of Cholera in Philadelphia, *believed to be, however, nothing more than the common cholera morbus.*"

The Medical Staff of Albany say in one of their reports, "although but few deaths took place from the 20th of June to the 3d of July, there was considerable sickness, and experienced physicians foresaw the coming danger in the *unusual prevalence of diarrhœa and common cholera morbus.*"

Dr. Rhinebeck, of N. Y., visited Canada for the purpose of witnessing the character of the Epidemic. On his return he addressed a letter to the New York Standard, under date of July 1st, in which he says: "The disease is Epidemic—it is atmospheric, and appears to obey the same laws as influenza. * * * Every person in these Provinces (Canada,) was affected by the same premonitory symptoms, viz: pain in the region of the stomach, a burning sensation in the bowels, and a fulness or expansion of the abdomen. *These feelings were universal, and I may safely say, not one person escaped them.*"

Dr. L. B. Beck, appointed, by Gov. Throop, General Health Officer, went to the frontiers on the north, to make farther inquiries in relation to Cholera. He wrote as follows:

WHITE HALL, July 14th, 1832.

To His Excellency Gov. Throop:

SIR:—I arrived here this morning, having visited on my way the Board of Health of Troy, Waterford, Schuylerville, Fort Edward and Fort Ann, and made such inquiries of the medical gentlemen of those places as I considered necessary to a complete history of the Cholera in this State. * * The information which I have received on my route seems to me *to prove conclusively, that the disease now prevailing at Albany, was not brought there by emigrants.* Complaints of a character similar to those which have prevailed so extensively in Albany for some weeks, *have been, and still are, equally general in all places which I have visited.* All other diseases appear to have been merged in this. Indeed, this is so much the case, that at Mechanicsville, Fort Ann, and White Hall, where intermittents have usually been very rife at this season of the year, few, if any, cases have occurred. Most of the physicians of whom I have made inquiries *have* (*had?*) *repeated cases of what they considered at the time unusual forms of common cholera morbus of the country.* They are characterized, in some instances, by severe and general spasms, and a coldness of the extremities—a peculiar sharpness of the features—violent pain in the region of the stomach—vomiting and purging of a limpid or nearly limpid fluid, and a more or less discoloration of the skin. *These cases have occurred among residents who were not known to have any intercourse with emigrants,* as well as among those who have been engaged on the canal."

From these, and a multitude of similar facts that might be adduced, we think, the following conclusions may be justly drawn:

*First*,—That the primary cause of Epidemic Cholera, was either solar, sidereal, lunar, atmospheric, electric, or telluric; operating generally on all persons within its sphere of action, but affecting different persons differently according to age, sex, temperament, habits, exposure, &c. The exciting causes were various; such as heat, filth, damp and crowded apartments, unwholesome food, intemperance, and the like.

*Second*.—That the Cholera was not imported—was not, therefore, Asiatic Cholera, which is a misnomer—but was indigenous, and was, strictly speaking, Cholera Asphyxia, which will appear more fully as we proceed in our enquiries.

*Third*.—That in no just sense of the term was it contagious—that it was not propagated by some virus generated *per se* and communicated from person to person, but by some subtle general agent, pervading the atmosphere, and extending over large districts of country.

*Fourth*.—That all Quarantine restrictions and regulations were not only useless, but injurious.

Respecting the diarrhœa which preceded, accompanied, and continued some time after the disappearance of fatal cases of Cholera, there were several peculiarities worthy of special notice.

*First*.—In most, if not all instances, it was attended by a sense of weight in the abdomen, most patients describing it as "a ball about the size of his two fists," which was always more sensibly felt after eating, and aggravated by a mis-step, or fall, received in walking.

*Second:* No case of Cholera occurred without this diarrhœa preceding it from three or four hours to three and four days, and sometimes weeks. We took particular pains to determine this fact, and we very much doubt whether that case has ever yet occurred without it. As the medical journals and public prints had not at that time mentioned, or dwelt on this important circumstance, but always spoke of attacks of Cholera as occurring suddenly, and lasting but a few hours, we were quite surprised when, in our own practice, we met with no such case; for we were prescribing for twenty cases of diarrhœa and cholera morbus, when we were for one of Cholera Asphyxia, and every case of Cholera was ushered in by a diarhœa of *several days' continuance;* hence, when we heard it reported in town that such and such a person was perfectly well, till such an hour, when he was suddenly seized with Cholera, and in six, eight or ten hours was dead, we forthwith went to the family, and inquired into the matter, and in every instance found the report erroneous. The mistake always arose from the fact, that the diarhœa being attended with very little inconvenience, the patient kept about his ordinary pursuits, and made no complaint till seized with vomiting and purging, which alone attracted attention, and was called Cholera, the diarrhœa being only *premonitory, no part of the disease,* and not worth mentioning, in the estimation of ordinary observers.

*Third:* The tongue had a thick, swollen appearance. The surface was covered with a third white, milky coat. The substance of it had a livid color.

*Fourth:* The pulse was preternaturally slow, ranging in adults from 40 to 60 per minute. This fact was verified by hundreds of observations. The slow pulse and livid tongue were noticed in many persons, who had no other symptoms of the Epidemic; suffered no inconvenience, and considered themselves in ordinary health.

*Fifth:* There were spasms of the muscles in the soles of the feet, calves of the legs and palms of the hands, also a cold clammy perspiration especially in the night when in bed. These symptoms, likewise existed in many instances without the diarrhœa.

*Sixth:* But the most important peculiarity noticed was in the quality of the blood itself. Even early in the season, when for any cause blood was drawn, it was observed to be unusually dark and heavy, and when allowed to cool, the coagulum was found to be disproportionately large, compared with the serum, and as the season and the Epidemic influence progressed, this disproportion and dark color, gradually increased. This fact, like the slow pulse, was more marked in decided cases of diarrhœa and Cholera, but was often very striking in persons, who had neither. These phenomena were common long before Cholera made its appearance.

If, to the foregoing, we add the appearances on dissection, after death by Cholera, we believe that we shall find a manifest and satisfactory solution of all the phenomena of both diarrhæa and Cholera asphyxia. In other words, we shall have the pathology of the disease, and consequently, the indications of *rational* treatment.

The appearances on dissection are not uniform, and there are, of course, discrepancies in the descriptions of different observers; but this is true of many other diseases. But in one great general fact all agree, viz:—The very great amount of congestion of the mesenteric and portal vessels, and those of the abdominal viscera. The chain of causes and effects seem, then, to be in the following order:—A subtle, unknown, morbific agent operated insensibly on the system for a greater or less period; slowly or more rapidly according to its intensity, rendering the blood thick, that is, destroying the normal proportion between the crassamentum and serum. Thus the circulation became more difficult and tardy; hence the slow pulse. But as the circulation became slower, of course the function of the lungs was performed less perfectly, and hence another cause of deterioration; the blood not being sufficiently relieved of its carbon, became still blacker and heavier. And thus the power to generate animal heat was impaired, and hence the cold and clammy skin. Up to this point, no link in the chain of causes seems wanting. But between those just enumerated, and that next, there is one or more lost. After the preceding symptoms have existed for an uncertain time, there begins, by degrees, to be congestion of the portal and mesenteric vessels, slight at first, but gradually increasing, and, as a consequence to this congestion, we have occasional pains in the abomen, borborygmus, uneasiness after eating, or sense of weight in the bowels, and a *livid swollen tongue.* But the congestion increases, and now we begin to have diarrhœa, when all the other symptoms are more or less aggravated. *This constitutes the first stage of Cholera,* and not the premonitory, as it was most erroneously called. It may continue longer or shorter, according to circumstances—as age, sex, habits, exposure, constitution or *treatment.* In hundreds of instances it will be not only the first, but last and only stage: the constitution being adequate to resist and overcome all further influence of the primary morbific agent of Cholera, and health be gradually restored. But if from predisposition, or mismanagement, or the more intense action of the Epidemic cause, this congestion proceeds to another degree, then, the serum, already too scanty, begins, in consequence of the more and more obstructed circulation in both the large and capillary vessels, to be forced, as it were, through the follicles of the mucous membrane, into the stomach and intestines, large and small. And now commences what has been termed *vomiting and purging.* This is the beginning of the second stage, which may last from three hours to thirty. All secretion of bile, urine, and even mucus, ceases. Absorption also is completely suspended. The blood now loosing its serum from every pore, becomes more black and thick; all power to generate animal heat seems to be extinct, and the surface of the body is as cold as if the patient were already dead, while within he seems to be on fire, and cries unceasingly, water! The third stage now rapidly supervenes—but at what precise point it begins, cannot well be defined. The dejections and ejections grow less frequent and profuse; but the skin becomes more livid and wrinkled; the features more pinched and ghastly—the breath cold; the voice cracked and husky; the pulse imperceptible; the action of the heart, a mere flutter till it ceases to beat altogether, and death closes the scene. Sometimes there is a fourth stage, or sequel to the third, or stage of collapse. The patient may survive this, and a feeble, imperfect kind of reaction takes place, and produce a species of typhus fever, which may run a longer or shorter course, and terminate sometimes in recovery, but oftener in death.

We have already intimated that what is called *vomiting* and *purging* in the second stage, is an erroneous view of the facts. The patient will deny to your queries, that he has either nausea or inclination to stool, and perhaps before the words are really out of his mouth, he will suddenly, without premonition, eject from his stomach and discharge from his bowels a large quantity of serous fluid, as if thrown by a force-pump. This seems to be rather the effect of a general spasmodic action of all the muscles of both the abdomen and viscera. For if you can induce *real nausea*, and a constant retching, these spasmodic discharges are greatly restrained, if not wholly arrested.

Our views of the pathology of Cholera Asphyxia are briefly these:—*First:* An altered condition of the blood, produced by some unknown agent, whereby the crassamentum is rendered too abundant in proportion to the serum, and too highly *carbonized;* and, *Second;* That extreme and general congestion of the portal and mesenteric vessels, and of the capillaries of the stomach and intestines is induced by the same, or some other unknown cause. These two conditions constitute the *essential* elements of Cholera Asphyxia, and are sufficient to account for all the successive symptoms. If these views are correct, what then are the indications of cure? Most assuredly to render the blood *thinner*, and to restore the equilibrium of the circulation, by removing the congestion of the abdominal viscera. What are the appropriate means to effect these objects?

Among those who take this view of the pathology, there can be no difference of opinion about remedies. We are aware that all kinds of treatment have been adopted, recommended, and defended; the narcotic, astringent, stimulant, alterative, cathartic, emetic, and depletory. *We have tried them all,* and seen them tried by others; but we truly and solemnly aver, that we never saw any satisfactory results from any, except the two last mentioned. If we are pointed to the success of the other methods, and to the failures of the latter, our answer is, that the vital powers of some constitutions will triumph sometimes over both disease and the worst treatment, while, on the other hand, there are cases so surrounded by untoward circumstances, as to defy all, and the only appropriate remedies, even when administered most skilfully. How can narcotics decarbonize the blood and relieve congestion? They produce these conditions when they do not exist, and aggravate them when they do. We apprehend the chloroform treatment will be found to have the same effect. With our present views we could hardly be induced to give it a trial. Small repeated anodynes may be useful adjuvants to other remedies by moderating pain and spasms. As to astringents, they may, in the first stage, somewhat restrain the discharges, and do no great injury, and in moderate cases the patient may recover. But how can astringents fulfil the indications already specified? It is plain they cannot relieve or remove the congestion; much less the algid condition of the blood.

Judging from the coldness of the surface, and the feebleness of the pulse we are strongly tempted to resort to stimulants. But no amount of stimulants, internal nor external, can restore the animal heat, while the blood grows thicker, and more and more carbonized. We have seen the surface nearly par-boiled by hot vapor, by means of heated bricks, and stimulants freely administered at the same time, yet not the least degree of warmth imparted to the body. It is evident that stimulants cannot, any more than astringents, fulfill the indications already stated. As to alteratives, there is no time for their action, unless commenced and persevered in during the stage of diarrhœa—for, during the second stage, all absorption is suspended, and for this reason, almost all internal remedies fail.

As to cathartics of all kinds, except calomel, we consider them absolutely injurious and highly dangerous. Salts and oil are among the very worst. The slightest cases of diarrhœa will be immediately converted into the worst forms of cholera, by small portions of either. This effect of cathartics is easily explained by the congested state of the visceral vessels.

But emetics and venesection, differ in their effect from all the other remedies mentioned. Yet so deceitful are appearances, that, if we are guided in our treatment *by symptoms only*, they are wholly contra-indicated. The profuse discharges, pain and spasms, seem to demand narcotics and astringents—the coldness, stimulants and rubefacients—the suspension of bile and other secretions, alteratives, &c., while the vomiting, prostration, faintness, feeble and rapid pulse, seem certainly to forbid the use of emetics and the lancet. Of all diseases "that flesh is heir to," there is probably none in which appearances are so deceitful; in which symptoms prove to be such a perfect "Ignis Fatuus" in treatment.

We have already said, that what has been called *vomiting* and *purging*, are, strictly speaking, no such thing, but a spasmodic action of the muscles of the abdomen, &c., which seem to be induced by some mysterious sympathy between the nervous system and the stomach and bowels, when they have become filled by the serum of the blood being poured into them, or by the congestion of the mesenteric vessels. Now, although emetics do not immediately change the quality of the blood, yet they are, by their mechanical action, a powerful means of relieving congestion of internal organs. They are also the most speedy *alteratives* in certain cases, for restoring or promoting the secretions. By the concussion of the abdominal muscles in vomiting, the large and deep seated blood vessels are compressed, and the circulation driven, as it were, to the surface, by a kind of centrifugal force. In 1832, Dr. Yates, I think, of New York, published a pamphlet, in which he solemnly averred that he treated a great many cases of cholera in that city; that he did not lose a single patient, and that his principal remedy was a solution of tart. antimo., combined with Tinct. opii, given in small repeated doses, till full vomiting was produced. We should prefer the vin. anti,, with tinct. opii. Our own experience, however, with emetics, is not extensive, yet we saw two of the most hopeless cases in the very last stage of collapse, speedily relieved by emesis: the one, by giving over doses of aqua ammoniæ, with a view of stimulating the patient, but which produced excessive nausea and retching, under which the pulse returned, the color of the lips was restored, and the patient completely cured; the other, by several table spoonsful of salt, given with the view of vomiting. But, as has been already remarked, the primary, appreciable link in the chain of morbid action, is the deterioration of the blood, and as emetics cannot speedily enough correct this evil, the abstraction of blood seems to fulfil, at once, all indications; and this, in some form, we consider the principal remedy in all stages of the severer cases of Cholera. Some modificatiod of mode, however, is required. We do not say that venesection is necessary in all cases, and that there may not be those where it is contra-indicated. In the first stage it is always safe, and we believe will not fail to cure, ninety-nine times in a hundred, and in many cases, be all that is needed, except caution in diet; the powers of nature will do the rest. But if other remedies are required, they will then operate kindly and efficiently, whereas, without venesection, they would irritate and aggravate, or prove nert.

In the second stage, owing to the loss of serum, the imperfect respiration and decarbonization of the blood, and its consequently thick and tar like condition, and therefore, the *asphyxied* state of the brain and nervous system, bleeding will be more difficult, and must be performed with more care. The blood will run slowly at first—it is best it should—but persevere till it flows freely, and till its color and consistency becomes redder and thinner. If it can not be drawn from the arm, open the temporal artery, or apply leeches or cups to the epigastrium. In the stage of collapse the indications are the same, and we must still endeavor to abstract blood. Stimulating emetics may aid us, by giving a temporary impetus to the circulation, till enough blood has flown to relieve the laboring lungs; the struggling heat and paralyzed brain. When this is accomplished, the generation of animal heat begins, the secretions and all other functions are restored. Of the *modus operandi* of venescetion, it is unnecessary to speak. It must be obvious to every intelligent Physician. For it is a well established fact that the abstraction of blood rapidly diminishes the proportion of crassamentum, and, if carried to excess, produces a dropsical condition, a state of things incompatible with Cholera. It was observed in 1832, that nursing and feeble women seldom had Cholera. We can *imagine* that lactation and menstuation, or menorrhagia, would so reduce the blood as to counteract the epidemic influence.

It would be easy to quote high authority for the practice of bleeding, but our article has grown much larger than we intended, and we will content ourselves therefore by referring to the report of Dr. Walker to the Gov. of Massachusetts, published in a late No. of the Boston Medical and Surgical Journal. Dr. W. had charge of the Massachusetts State's Prison, when the Cholera suddenly broke out, and 115 convicts sickened in 24 hours; 196 had the disease, and one of the whole number died. We have never seen so concise, and lucid a detail of treatment as given in this report. The treatment itself we consider a perfect model. For promptness, and *strait forwardness*, we think it, worthy of all praise. We must quote one paragraph. He says; "When practised at the commencement of the disease, bleeding was followed by *immediate relief*. So much so, that men with *skin and tongue cold and pulse absent, or scarcely perceptible*, were entirely relieved by the loss of from sixteen to thirty two ounces of blood; the pain was alleviated, vomiting and diarrhœa removed, and the disease, as it were, *extinguished*."

With regard to external applications our experience is against them all, whether sinapisms, cataplasms, rubefacients, hot baths, fomentations, *et omne id genus* They are exceedingly disagreeable to the patient, increase his restlessness, fatigued, and debilitated him, without affording any adequate compensation. Friction with dry flannel is all that we permit.

Besides the emetics combined with laudanum, we use, as an anodyne, alterative, and stimulant, after bleeding, a combination of opium, camphor, capsicum and calomel, varied in proportion and quantity according as circumstances seem to demand; say opium gr. ½ to 1, camph. gr. 1, cap. gr. 1 to 5, and calomel gr. 2 to 5; repeated every half hour, hour, two hours, &c. Instead of cold drinks, give small pieces of ice, which are more grateful to the patient than anything else.

We must now be permitted to indulge a little in speculation; for hitherto we have endeavored to keep as close to facts as possible. Cholera Asphyxia has again invaded our country, and many believe that it has been *imported.* We believe no such thing. During the last summer, dysentery prevailed throughout the northern states. That dysentery was accompanied in many instances by unusual serous discharges—and many cases terminated fatally, not so much from the usual bloody mucous discharges, as by serous ones. We remarked to others more than once, that patients with these evacuations had very much the general appearance as those with Cholera. The dysentery, too, like Cholera, was greatly aggravated by cathartics, and those physicians who did not make this discovery, have a fearful accountability to answer for. We know, too, that this disease, like Cholera, was *always* cured by full bleeding in the early stage of it. In short we believe that a *choleric* influence has existed in this region of country for a year. Again, although dysentery disappeared about the first of October, and the months of November and December, now past, were remarkably healthy, yet there have occurred, and are now occurring every week, cases of diarrhœa, and even of cholera morbus in our city and county. Six cases of severe vomiting and purging have come to my knowledge within two weeks. I therefore apprehend that we are already in the forming stage of the Epidemic influence. What that influence is we may never know. Among the known agents, telluric electricity seems to be the most probable. It may however be some agent not yet known to human science.

On the subject of prophylactics, we would make a suggestion, for consideration and observation. While we know not the primary cause of Cholera, we cannot hope to destroy that cause; we can only aim to counteract its deleterious influence on the system. If our view of the pathology of this disease be the true one, then it follows, that, besides "temperance in all things," a system of dieting that will reduce the crassamentum of the blood is the proper one. Now this we can do by a liberal use of acetic acid. The most agreeable and convenient articles are vinegar as a condiment, and cider as a beverage. We came to this conclusion by a process rather "a priori," and, therefore, only offer it as a speculation. The only fact we have on which to base this opinion, is the well known effect of vinegar, if used to excess, in producing a pale skin and general debility. And why may not acetic acid be as useful in Cholera, as the tartaric and citric in preventing and curing scurvy? We offer the suggestion only for what it is worth.

ROCHESTER, N. Y., Jan. 5, 1849.

